# Supplementary material for: Association of Kawasaki disease with urbanization level and family characteristics in Taiwan: A nested case–control study using national-level data
Source: PLoS One. 2024 Jan 5;19(1):e0296505. doi: 10.1371/journal.pone.0296505 (PMC10769101; doi:10.1371/journal.pone.0296505)
Supplement: Attachment — Submitted filename: Response Letter.doc [file pone.0296505.s002.doc]

Dong Keon Yon, MD

Academic Editor

PLOS ONE

Oct. 23 2023

Dear Dr. Dong Keon Yon:

Re: Document reference No. PONE-D-23-14951

Please find attached a revised version of our document “**Association of Kawasaki disease with urbanization level and family characteristics** **in Taiwan: A nested case–control study using national-level data**”. We would like to resubmit for publication as a original article in PLOS ON

Your comments and those of the reviewers were highly insightful and enabled us to improve the quality of our document. In the following pages are our responses to each comment from the reviewer(s) as well as your own comments.

Revisions in the text are shown blue highlights. We hope that our revisions to the document combined with our accompanying responses will be sufficient to render our document suitable for publication in PLOS ONE.

We look forward to hearing from you soon.

Yours sincerely,

Chih-Jung Yeh

School of Public Health, Chung Shan Medical University

Tel.: 886 4 24730022 #12183

Fax: +886 4 23248179

E-Mail: alexyeh@csmu.edu.tw

Address: No. 110, Sec. 1, Jianguo N. Rd., South District, Taichung City 40201, Taiwan.

James Cheng-Chung Wei

Department of Allergy, Immunology & Rheumatology, Chung Shan Medical University Hospital

Tel.: +886 4 24739595 #34718

Fax: +886 4 24637389

E-Mail: jccwei@gmail.com

Address: No. 110, Sec. 1, Jianguo N. Rd., South District, Taichung City 40201, Taiwan.

**Responses to the comments of Editor-in-Chief (If any)**

\# All associations were considered statistically significant when P values were <.05. -> All associations were considered statistically significant when two-sided P values were <.05.

Reply:

Thank you for your correction. We modified the original sentence of ‘All associations were considered statistically significant when P values were <.05.’ to 'All associations were considered statistically significant when two-sided P values were < .05.'

Modified sentence:

Line 142-144: “All statistical analyses were performed using SAS 9.4 (SAS Institute, Cary, NC, USA). A two-sided *P* value of <.05 indicated statistical significance.”

\# We constructed the conditional logistic regression model to explore the association between baseline parental allergic disease and the risk of KD in children before the age of 5 years. -> Please cite the statistical paper (DOI: https://doi.org/10.54724/lc.2022.e3).

Reply:

Thank you very much for your suggestion. We added the reference (DOI: https://doi.org/10.54724/lc.2022.e3) to explain the regression analysis method.

Modified sentence:

Line 136-138: We constructed the conditional logistic regression model to explore the association between baseline parental allergic disease and the risk of KD in children before the age of 5 years [18].

**Responses to the comments of Reviewer #1**

Reviewer #1: This paper presents an investigation of the association of Kawasaki disease with urbanisation level and family characteristics in Taiwan using multi-dataset. The findings are interesting and may contribute to existing literature. The following issues need to be clarified before this reviewer has a favourable recommendation.

1. The latest NHIRD available would be for year 2021 or at least 2020. Please clarify why the latest data were not included, and how this is likely to impact the results drawn.

Reply: We initiated the application for NHIRD data authorization to the Health and Welfare Data Science Center in 2019, and the requested data covers the period from 2003 to 2017. NHIRD has been updated to the year 2021. However, based on our initial project proposal and the feasibility of data analysis, we are unable to utilize this latest data for our analysis. Future research can focus on analyzing the updated data to provide more meaningful scientific insights.

Modified sentence:

Line 251-253: “Third, the NHIRD has been updated until 2021. However, based on our initial project proposal and the feasibility of data analysis, we are unable to utilize the latest data.”

2. Confusion: in line 67, authors pointed out that the datasets used range from 2006 to 2015; however, in line 72, it was for 2003-2017.

Reply: Sorry for the confusion, the wording was not clear and led to your misunderstanding. We selected the children born in Taiwan between January 1, 2006, and December 31, 2015, (n = 1 939 449) and observed their KD development before the age of 5 years. The current study utilized three nationwide databases in Taiwan, namely the NHIRD, the NBRD, and the National Death Index Database (NDID); Data from the NHIRD spanning from 2003 to 2017, the NBRD from 2006 to 2015, and the NDID from 2006 to 2017 were requested to identify baseline characteristics, including demographics, parental allergic diseases, and perinatal conditions, as well as the development of Kawasaki disease in offspring.

Modified sentence:

Line 65-67: “For this retrospective nested case–control study, we selected children born in Taiwan between January 1, 2006, and December 31, 2015, (n = 1,939,449) and observed the development of KD before the age of 5 years in these children.”

and

Line 69-73: “We used three nationwide databases: the NHIRD (from 2003 to 2017), NBRD (from 2006 to 2015), and National Death Index Database (NDID, from 2006 to 2017). From the databases, baseline characteristics, namely demographic characteristics, parental allergic diseases, and perinatal conditions, and KD development in offspring, were obtained”

3. It would be informative to provide readers who are not familiary the datasets (esp. NHIRD) with characteristics of the datasets. A vast majority of population is covered?

Reply: Many thanks for your valuable suggestion, we added the information of datasets in line 73-78.

Modified sentence:

Line 73-78: “The NHIRD is one of the largest and most comprehensive healthcare databases in the world [13] and includes medical claims data of >99% of Taiwan residents. Data on medical claims for inpatient and outpatient visits to clinics or hospitals (diagnosis codes and date of visit) were obtained from the NHIRD [13]. The incidence of maternal diseases (hypertension and diabetes) and childhood KD was obtained by reviewing medical claim records.”

4. What is the reason(s) for cutpoints for some variables such as maternal age at delivery/father's age at delivery?

Reply: Several studies have shown that advanced maternal age (35 years or older) is associated with higher risks of maternal morbidity, obstetric interventions, and adverse pregnancy outcomes (CMAJ. 2008 Jan 15; 178(2): 165–172). Previous research has also demonstrated that children born to mothers under the age of 25 or over the age of 35 exhibit poorer outcomes in terms of mortality, self-reported health, height, obesity, and the number of diagnosed conditions compared to those born to mothers aged 25–34 (Demography. 2012 Nov; 49(4): 10.1007/s13524-012-0132-x). Therefore, we categorized maternal/paternal age into four groups: <25, 25-34, 35-45, and ≥45 years old. The reasons for this categorization are provided in lines 81-87.

Modified sentence:

Line 81-87: “Advanced maternal age (≥35 years) has been associated with higher risks of maternal morbidity, obstetric interventions, and adverse pregnancy outcomes[14]. A study demonstrated that children born to mothers aged <25 or >35 years had poorer outcomes in terms of mortality, self-reported health, height, obesity, and the number of diagnosed conditions than those born to mothers aged 25–34 years [15]. Therefore, we categorized maternal/paternal age into four groups: <25, 25–34, 35–45, and ≥45 years old.”

5. Was the multi-collinearity problem assessed? It seems to this reviewer that several comorbidities may exist at the same time and they may be stronly correlated.

Reply: We have evaluated the multicollinearity problem in the logistic regression analysis. The regression model does not exhibit severe multicollinearity. In Table 2, we included the crude odds ratios (OR) for offspring's Kawasaki disease associated with various parental comorbidities, as well as the adjusted odds ratios (aOR) after controlling for baseline demographics and perinatal conditions. Although the crude OR results indicate significant associations between paternal allergic rhinitis, hypertension, hyperlipidemia, chronic liver disease, and offspring's Kawasaki disease, all the aOR values are similar to the crude OR. This also suggests there is a very mild multicollinearity problem when estimating the association between offspring's Kawasaki disease and paternal comorbidities.

Modified sentence:

1.We added the crude ORs in Table 2.

**Table 2. Results of Conditional Multivariable Logistic Regression Analysis for Risk Factors Among Children with Kawasaki Disease**

| **Factor** | **Control Group (N= 59 809)** | **Kawasaki (N= 7566)** | **Crude OR**  **(95% CI)** | **Adjusted OR**  **(95% CI)** |
| --- | --- | --- | --- | --- |
| **Allergy disease** |  |  |  |  |
| **Atopic dermatitis** |  |  |  | p for interaction=0.2668 |
| **Mother** | 1119(1.87%) | 141(1.86%) | 0.996(0.835-1.189) | 0.991(0.828-1.186) |
| **Father** | 303(0.51%) | 37(0.49%) | 0.965(0.686-1.359) | 0.905(0.630-1.300) |
| **Allergic rhinitis** |  |  |  | p for interaction=0.7031 |
| **Mother** | 5044(8.43%) | 647(8.55%) | 1.015(0.932-1.106) | 1.003(0.913-1.102) |
| **Father** | 4536(7.58%) | 622(8.22%) | 1.092(1.000-1.191) | 1.072(0.973-1.181) |
| **Asthma** |  |  |  | p for interaction=0.3905 |
| **Mother** | 871(1.46%) | 123(1.63%) | 1.118(0.924-1.353) | 1.089(0.893-1.329) |
| **Father** | 834(1.39%) | 100(1.32%) | 0.947(0.769-1.167) | 0.896(0.719-1.117) |
| **Hypertension** |  |  |  | p for interaction=0.2078 |
| **Mother** | 606(1.01%) | 83(1.10%) | 1.048(0.891-1.234) | 1.127(0.882-1.441) |
| **Father** | 1642(2.75%) | 238(3.15%) | 1.151(1.002-1.321) | 1.137(0.987-1.309) |
| **Hyperlipidemia** |  |  |  | p for interaction=0.5155 |
| **Mother** | 385(0.64%) | 42(0.56%) | 0.862(0.626-1.186) | 0.873(0.620-1.229) |
| **Father** | 2025(3.39%) | 294(3.89%) | 1.154(1.018-1.307) | 1.125(0.991-1.277) |
| **Chronic liver disease** |  |  |  | p for interaction=0.4180 |
| **Mother** | 1268(2.12%) | 168(2.22%) | 1.048(0.891-1.234) | 1.044(0.872-1.251) |
| **Father** | 4146(6.93%) | 575(7.60%) | 1.104(1.009-1.209) | 1.085(0.988-1.192) |
| **Diabetes** |  |  |  | p for interaction=0.6786 |
| **Mother** | 690(1.15%) | 76(1.00%) | 0.869(0.685-1.103) | 0.850(0.666-1.085) |
| **Father** | 693(1.16%) | 90(1.19%) | 1.027(0.823-1.281) | 1.028(0.821-1.288) |
| **COPD** |  |  |  | p for interaction=0.3199 |
| **Mother** | 241(0.40%) | 35(0.46%) | 1.149(0.805-1.639) | 1.065(0.730-1.554) |
| **Father** | 387(0.65%) | 52(0.69%) | 1.063(0.795-1.421) | 1.001(0.740-1.355) |
| **Autoimmune disease** |  |  |  |  |
| **Diffuse diseases of connective tissue** |  |  |  | p for interaction=0.9299 |
| **Mother** | 341(0.57%) | 43(0.57%) | 0.997(0.725-1.370) | 0.971(0.705-1.336) |
| **Father** | 86(0.14%) | 10(0.13%) | 0.919(0.477-1.770) | 0.957(0.495-1.853) |
| **Rheumatoid arthritis and other inflammatory polyarthropathies** |  |  |  | p for interaction=0.9403 |
| **Mother** | 70(0.12%) | 11(0.15%) | 1.243(0.658-2.347) | 1.230(0.649-2.331) |
| **Father** | 67(0.11%) | 13(0.17%) | 1.535(0.847-2.781) | 1.530(0.842-2.782) |
| **Ankylosing spondylitis and other inflammatory spondylopathies** |  |  |  | p for interaction=0.9192 |
| **Mother** | 101(0.17%) | 14(0.19%) | 1.096(0.626-1.918) | 1.105(0.630-1.938) |
| **Father** | 329(0.55%) | 39(0.52%) | 0.937(0.672-1.307) | 0.920(0.659-1.285) |
| **Depressive disorder** |  |  |  | p for interaction=0.1597 |
| **Mother** | 1714(2.87%) | 213(2.82%) | 0.982(0.850-1.134) | 1.017(0.875-1.182) |
| **Father** | 1534(2.56%) | 198(2.62%) | 1.021(0.879-1.186) | 1.058(0.905-1.237) |

2. Line 185-187: “The crude ORs indicated significant associations of paternal allergic rhinitis, hypertension, hyperlipidemia, and chronic liver disease with offspring KD, and the values of the adjusted ORs were similar to the crude ORs”.

**Responses to the comments of Reviewer #2**

Reviewer #2: This is a nation wide study of Kawasaki disease in Taiwan to investigate the cause of the disease. A family history of allergy, higher household income, smaller family size, and urbanization at birth were already reported in associated with incidence of KD. This paper confirmed the previous reports of Kawasaki disease. I agree the hygiene hypothesis to some extent, but your study did not mention about coronary involvement of the Kawasaki disease. I understand that this type of the study was very difficult to study the cause of coronary involvement, but please discuss the possible mechanism about higher maternal age and CAL.

Replay: Thank you to reviewers for affirming the conclusions of our study. We did not discuss about coronary artery invasion in Kawasaki disease in our study. This was not included in this designed experiment. In line 223-225 of our article: Huang et al. reported that maternal age less than 32 years may lead to fewer offspring with KD (Pediatr Rheumatol Online J. 2019;17(1):46. doi: 10.1186/s12969-019-0348-z). A higher maternal age is associated with a higher rate of KD with CAL and intravenous immunoglobulin resistance.

In previous paper (Pediatr Rheumatol Online J. 2019;17(1):46. doi: 10.1186/s12969-019-0348-z) described that “KD is reported to be a disease with autoimmune-like or autoinflammatory response that is triggered in genetically predisposed subjects by certain environmental factors. Growing evidence has shown a link between KD and immune-mediated diseases, especially allergic diseases or autoimmune diseases. Children with allergic diseases like urticaria, allergic rhinitis, and atopic dermatitis are subsequently at an increased risk of KD. In contrast, a subsequent risk for allergic diseases including asthma, allergic rhinitis, and atopic dermatitis has been found to be increased in patients with a history of KD. As for immune-mediated diseases, previous studies have indicated associations with increasing maternal age at delivery with an increased risk for type 1 diabetes and food allergies in the offspring. A fine-tuned balance between genetic, immunological, metabolic, and hormonal factors is necessary for reproduction, and all of these factors are likely involved in the aging process. Therefore, maternal aging may affect health outcomes in offspring, including with regard to the development of their immune response. “

I agree the above possible mechanism, of course, I also spent a lot of time trying used “Pubmed of national library medicine” search for the possible mechanism about high maternal age and KD with CAL. But I did not figure out other possible mechanism.

Modified sentence:

Line:228-230: “Huang et al. suggested that this may be due to the maternal aging process, which may affect the development of offspring immune response [6]”
