# Supplementary material for: Association of Kawasaki disease with urbanization level and family characteristics in Taiwan: A nested case–control study using national-level data
Source: PLoS One. 2024 Jan 5;19(1):e0296505. doi: 10.1371/journal.pone.0296505 (PMC10769101; doi:10.1371/journal.pone.0296505)
Supplement: Attachment — Submitted filename: reply to reviewer comment - 20231211.docx [file pone.0296505.s003.docx]

Dong Keon Yon, MD

Academic Editor

PLOS ONE

Oct. 23 2023

Dear Dr. Dong Keon Yon:

Re: Document reference No. PONE-D-23-14951

Please find attached a revised version of our document “**Association of Kawasaki disease with urbanization level and family characteristics** **in Taiwan: A nested case–control study using national-level data**”. We would like to resubmit for publication as a original article in PLOS ON

Your comments and those of the reviewers were highly insightful and enabled us to improve the quality of our document. In the following pages are our responses to each comment from the reviewer(s) as well as your own comments.

Revisions in the text are shown blue highlights. We hope that our revisions to the document combined with our accompanying responses will be sufficient to render our document suitable for publication in PLOS ONE.

We look forward to hearing from you soon.

Yours sincerely,

Chih-Jung Yeh

School of Public Health, Chung Shan Medical University

Tel.: 886 4 24730022 #12183

Fax: +886 4 23248179

E-Mail: alexyeh@csmu.edu.tw

Address: No. 110, Sec. 1, Jianguo N. Rd., South District, Taichung City 40201, Taiwan.

James Cheng-Chung Wei

Department of Allergy, Immunology & Rheumatology, Chung Shan Medical University Hospital

Tel.: +886 4 24739595 #34718

Fax: +886 4 24637389

E-Mail: jccwei@gmail.com

Address: No. 110, Sec. 1, Jianguo N. Rd., South District, Taichung City 40201, Taiwan.

**Responses to the comments of Reviewer**

6. Review Comments to the Author

Please use the space provided to explain your answers to the questions above. You may also include additional comments for the author, including concerns about

dual publication, research ethics, or publication ethics. (Please upload your review as an attachment if it exceeds 20,000 characters)

Reviewer #1: The authors did a good job by responding to this reviewer's previous comments and questions. My final question/comment is that the latest NHIRD was not analysed. Please clarify, in the respect of Kawasaki disease with urbanization level and family characteristics in Taiwan, how this is likely to impact the results drawn.

Reply: One limitation of our study is that we utilized NHIRD data spanning from 2003 to 2017, while NHIRD has been updated to include data up to 2021. Over the years, medical technology has made significant strides in Taiwan, leading to more accurate diagnoses of Kawasaki disease. Additionally, the declining total fertility rate has resulted in a higher proportion of single-child families, which could potentially contribute to an increased prevalence of Kawasaki disease. Furthermore, the outbreak of COVID-19 may also impact the prevalence of Kawasaki disease. However, due to the scope of our data ending in 2017, we were unable to evaluate the influence of these factors. Future research should consider utilizing the most recent data to assess the effects of these factors on the risk of Kawasaki disease.

Modified sentence:

Line 252-259 “Medical technology has advanced considerably over the years in Taiwan, leading to more accurate diagnoses of Kawasaki disease. Additionally, the declining overall fertility rate in Taiwan has resulted in an increased proportion of families with only one child, and this shift in family structure may contribute to an increased prevalence of Kawasaki disease. The COVID-19 outbreak may have also affected the prevalence of Kawasaki disease. However, we could not evaluate the influence of these factors because our data only covered up to the year 2017. Accordingly, future research should consider using the most updated data to assess the effects of these factors on the risk of Kawasaki disease..”

Reviewer #2: This is a nationwide study of Kawasaki disease. This manuscript is well revised. Please change Yoshio (Line 209) to Nagao in the text.

Reply: We greatly appreciate your valuable suggestion. We have made the necessary change from "Yoshio" to "Nagao" in line 209 to ensure the accuracy of the information presented. Once again, we sincerely thank you for your valuable input.

Modified sentence:

Line 209:” Yoshiro’s Nagao’s study revealed that the total fertility rate is negatively associated with the incidence of KD”
